# Insect Wing Membrane Topography Is Determined by the Dorsal Wing Epithelium

**DOI:** 10.1534/g3.112.004028

**Published:** 2013-01-01

**Authors:** Andrea D. Belalcazar, Kristy Doyle, Justin Hogan, David Neff, Simon Collier

**Affiliations:** Department of Biological Sciences, Marshall University, Huntington, West Virginia 25755

**Keywords:** Drosophila, hymenoptera, planar cell polarity, Prickle, Frizzled

## Abstract

The Drosophila wing consists of a transparent wing membrane supported by a network of wing veins. Previously, we have shown that the wing membrane cuticle is not flat but is organized into ridges that are the equivalent of one wing epithelial cell in width and multiple cells in length. These cuticle ridges have an anteroposterior orientation in the anterior wing and a proximodistal orientation in the posterior wing. The precise topography of the wing membrane is remarkable because it is a fusion of two independent cuticle contributions from the dorsal and ventral wing epithelia. Here, through morphological and genetic studies, we show that it is the dorsal wing epithelium that determines wing membrane topography. Specifically, we find that wing hair location and membrane topography are coordinated on the dorsal, but not ventral, surface of the wing. In addition, we find that altering Frizzled Planar Cell Polarity (*i.e.*, Fz PCP) signaling in the dorsal wing epithelium alone changes the membrane topography of both dorsal and ventral wing surfaces. We also examined the wing morphology of two model Hymenopterans, the honeybee *Apis mellifera* and the parasitic wasp *Nasonia vitripennis*. In both cases, wing hair location and wing membrane topography are coordinated on the dorsal, but not ventral, wing surface, suggesting that the dorsal wing epithelium also controls wing topography in these species. Because phylogenomic studies have identified the Hymenotera as basal within the Endopterygota family tree, these findings suggest that this is a primitive insect character.

Insects of the superorder Endopterygota form a wing from an epithelial sheet called a wing imaginal disc. The wing disc is a derivative of the embryonic ectoderm that continues to grow and develop throughout larval and pupal life. During pupal metamorphosis, a “wing pouch” within the disc folds out (everts) to generate an epithelial bilayer. The bilayer consists of dorsal and ventral epithelia that are attached at their basal surfaces and secrete wing cuticle from their apical surfaces. During wing maturation, dorsal and ventral epithelial cells are lost, leaving an adult wing structure that consists largely of inert cuticle. In the dipteran Drosophila, wing epithelial cells delaminate (via an epithelial to mesenchymal transition) shortly after the adult emerges from the pupal case and migrate out of the wing into the thorax (Kiger *et al.* 2007). The dorsal and ventral cuticle then fuse to form the mature wing membrane.

The wing membrane typically is decorated with structures, such as hairs or scales, and is normally supported by a network of wing veins. The specific pattern of veins determines much of the specific rigidity and flexibility required for the wing to function as a flight organ (Wootton 1992). In addition, the membrane itself can have a characteristic topography that also may contribute to wing flexibility. For example, we have previously shown that the Drosophila wing membrane is characterized by parallel ridges that are the equivalent of one epithelial cell in width (approximately 10 μm), and multiple cells in length wing [see Figure 1, B−D (Doyle *et al.* 2008; Valentine and Collier 2011)]. These ridges have an anteroposterior orientation in the anterior wing and a proximodistal orientation in the posterior wing.

**Figure 1:**
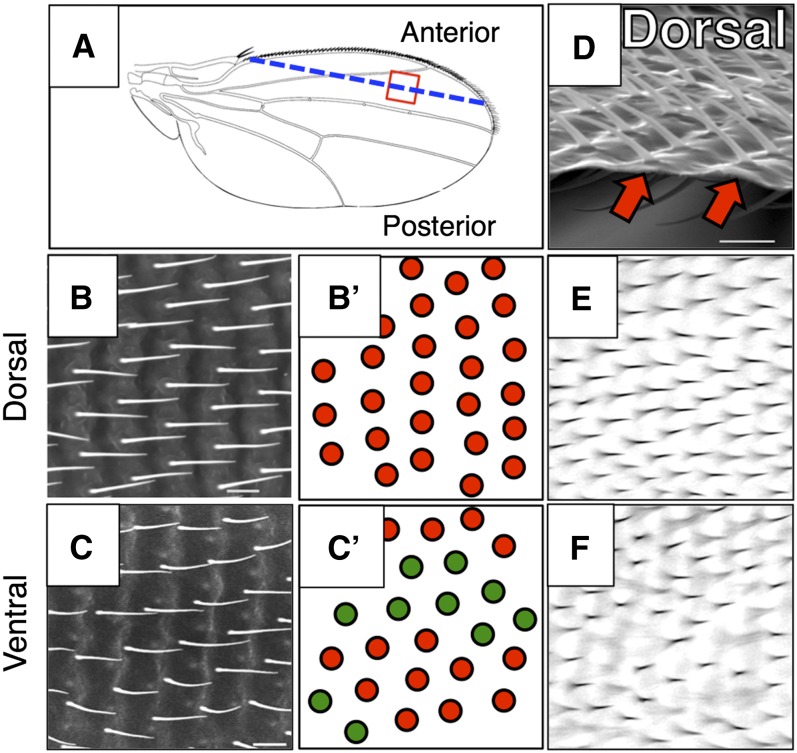
Hair positioning is coordinated with membrane topography on the dorsal but not ventral surface of the Drosophila wing. In panels B′ and C′; red circles represent wing hairs that are positioned at, or near, the top of membrane ridges and green circles represent hairs that are positioned at, or near, the bottom of membrane troughs. (A) Illustration of Drosophila dorsal wing; distal is to the right. The unshaded red square indicates the wing region imaged in panels B, C, E and F, and also in Figure 2; the blue dashed line represents the approximate incision made for the cut wing shown in panel D. (B) SEM of the dorsal surface of a Drosophila wing. (B′) Interpretation of panel B; all dorsal hairs are positioned at the tops of wing membrane ridges. (C) SEM of the ventral surface of a Drosophila wing. (C′) Interpretation of panel C; ventral hairs are located both on membrane ridges and within membrane troughs. (D) SEM of cut edge of Drosophila wing viewed from anterior, distal is left. Red shaded arrows indicate dorsal hairs located at the top of membrane ridges. (E) Light micrograph of hairs on the dorsal surface of a Drosophila wing. All dorsal hairs are visible in the same focal plane. (F) Light micrograph of hairs on ventral surface of a Drosophila wing. Only a subset of ventral hairs is visible in this particular focal plane.

The Drosophila wing membrane has an approximately uniform thickness of just a few hundred nanometers, which implies that the dorsal and ventral wing cuticle ultimately acquire complementary shapes despite being secreted from different sets of cells. This finding suggests either that the morphogenesis of dorsal and ventral cuticle is precisely coordinated or that the membrane’s topography is controlled primarily by just one wing epithelium (dorsal or ventral). In this report, we describe a combination of morphological and genetic studies that lead us to conclude that the topography of the Drosophila wing membrane is determined by the dorsal wing epithelium. A morphological analysis of wings from Hymenopterans, recently proposed to be a basal group within the Endopterygota (Savard *et al.* 2006), suggest that this is a primitive insect character.

## Methods

### Insect culture

*Drosophila melanogaster* used in this study were raised at 25° in standard yeasted cornmeal medium. Drosophila strains used were: *Oregon R* (Wild-type), *C765-Gal4* (*P{GawB}C-765*), *ap-Gal4* (*P{GawB}apmd544*) (Bloomington Stock Center), and *UAS-Sple* (David Gubb). Honey bee (*Apis mellifera*) wings were dissected from dead specimens provided by a local beekeeper (Samuel F. Kilgore). The parasitic wasp *Nasonia vitripennis* was obtained from the Carolina Biological Supply Company, or from Jack Werren (University of Rochester), as parasitized blowfly (Sarcophaga) pupae that were incubated at 30° until adult wasps emerged.

### Insect wing microscopy

#### Light microscopy:

Fly and bee wings were mounted in GMM (1 part methylsalicylate/1 part Canada balsam) for light microscopy. Wasp wings were mounted in air beneath a sealed cover slip.

#### Scanning electron microscopy:

Our scanning electron microscopy (SEM) protocols have been described previously (Doyle *et al.* 2008). Insects were anesthetized or killed, before we removed wings. Wings were attached to a JEOL standard aluminum stub (Electron Microscopy Sciences) using either colloidal graphite in isopropanol (Electron Microscopy Sciences) or standard nail polish. The specimen was then sputter-coated (Hummer 6.2 Plasma Coater) with approximately a 12-nm gold–palladium coat and imaged at 20 kV in a JEOL-5310LV SEM.

#### Cuticle refraction microscopy:

Our cuticle refraction microscopy (CRM) imaging protocol has been described in detail previously (Doyle *et al.* 2008; Neff *et al.* 2012). In brief, adult Drosophila wings were removed and laid gently on top of a thin layer of clear nail polish. The nail polish was allowed to dry and then a cover slip was placed on top and sealed with additional nail polish. Wings were viewed using an Olympus BX51 microscope (Olympus America Inc.) with the top lens of the condenser removed from the light path and the aperture diaphragm at its narrowest.

## Results and Discussion

Our interest in the role of the Frizzled Planar Cell Polarity (Fz PCP) signaling in controlling Drosophila wing membrane topography (Doyle *et al.* 2008) led us to study adult wing morphology using SEM. Using this approach, we observed that on the dorsal wing membrane, the wing hairs, which denote the apical center of epithelial cells, are consistently located at the tops of membrane ridges (Figure 1, B, B′, and D). Consequently, when viewed by light microscopy, dorsal wing hairs appear in the same focal plane (Figure 1E). However, ventral hairs are not consistently positioned with respect to membrane topography (Figure 1, C and C′), and so, under light microscopy, ventral hairs appear in differing focal planes (Figure 1F). This coordination of hair position and membrane topography on the dorsal, but not the ventral, wing surface raised the possibility that the dorsal wing epithelium controls wing membrane topography.

**Figure 2:**
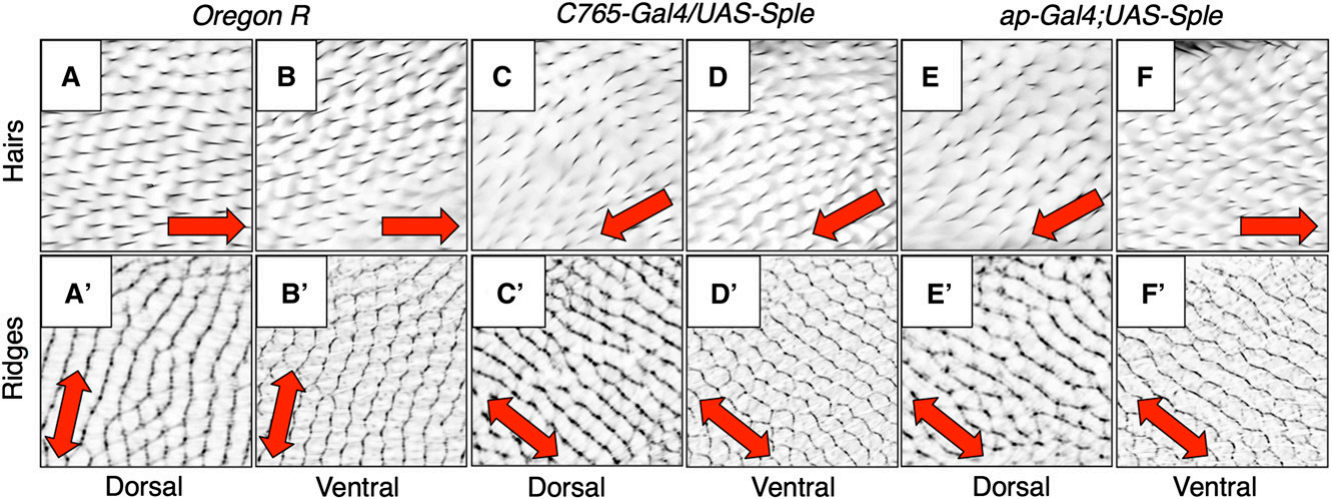
Drosophila wing membrane topography is controlled by the dorsal wing epithelium. Panels A-F are light micrographs of wing hairs in a region of the anterior wing represented by the unshaded red square in Figure 1A. Single-headed red arrows indicate local hair polarity. Panels A′-F′ are inverted CRM images showing the same region of the same wings shown in panels A-F, respectively. Double-headed red arrows indicate local ridge orientation. Genotype and wing surface imaged are as follows: (A, A′) *Oregon R* (wild-type) dorsal wing; (B, B′) *Oregon R* ventral wing; (C, C′) *C765-Gal4/UAS-sple* dorsal wing; (D, D′) *C765-Gal4/UAS-sple* ventral wing; (E, E′) *ap-Gal4;UAS-sple* dorsal wing; and (F, F′) *ap-Gal4;UAS-sple* ventral wing.

To facilitate our studies of Drosophila wing topography, we developed a technique (CRM) that allows us to visualize Drosophila wing membrane ridges using light microscopy (Doyle *et al.* 2008; Neff *et al.* 2012). Using the CRM method, we found that membrane ridges are visible in light micrographs as bright lines against a dark background. However, for clarity, we normally color-invert our CRM images so that ridges appear as dark lines on a light background (for example, see Figure 2A′). We have previously shown that the orientation of ridges on the Drosophila wing membrane is controlled by the Fz PCP signaling pathway (Doyle *et al.* 2008), which also controls wing hair polarity. Altering Fz PCP signaling in the wing can, therefore, change the orientation of both ridges and hairs. For example, uniform overexpression of the Sple isoform of the Prickle protein (*i.e.*, pk-PC), a component of the Fz PCP pathway (Gubb *et al.* 1999), alters hair and ridge orientation on both the dorsal and ventral wing (Doyle *et al.* 2008). Normally, in the anterior wing, dorsal and ventral hairs point distally (Figure 2, A and B), and dorsal and ventral ridges have an anteroposterior orientation (Figure 2, A′ and B′). Uniform overexpression of the Sple isoform in both dorsal and ventral wing epithelia (using the *C765-Gal4* driver in combination with a *UAS-sple* transgene) generates reproducible changes in hair and ridge orientation on both dorsal and ventral wing surfaces. Hair polarity appears rotated approximately 150° clockwise (Figure 2, C and D), and ridges appear rotated approximately 60° counterclockwise (Figure 2, C′ and D′). To test our hypothesis that the dorsal epithelium determines wing membrane topography, we overexpressed the Sple isoform in the dorsal wing epithelium alone by using the *apterous-Gal4* (*ap-Gal4*) driver. We hypothesized that Sple expression in the dorsal epithelium would alter hair polarity on the dorsal but not ventral wing surface but would change ridge orientation on both surfaces. Indeed, Sple overexpression in the dorsal epithelium rotates dorsal hair polarity approximately 150° clockwise (Figure 2E), but ventral hair polarity remains wild-type (Figure 2F). However, both dorsal and ventral wing surfaces display the 60° counterclockwise ridge rotation typical of uniform Sple over-expression (Figure 2, E′ and F′). The fact that Sple overexpression in the dorsal epithelium alone alters membrane topography on both dorsal and ventral wing surfaces supports our hypothesis that the dorsal epithelium controls Drosophila wing membrane topography.

**Figure 3:**
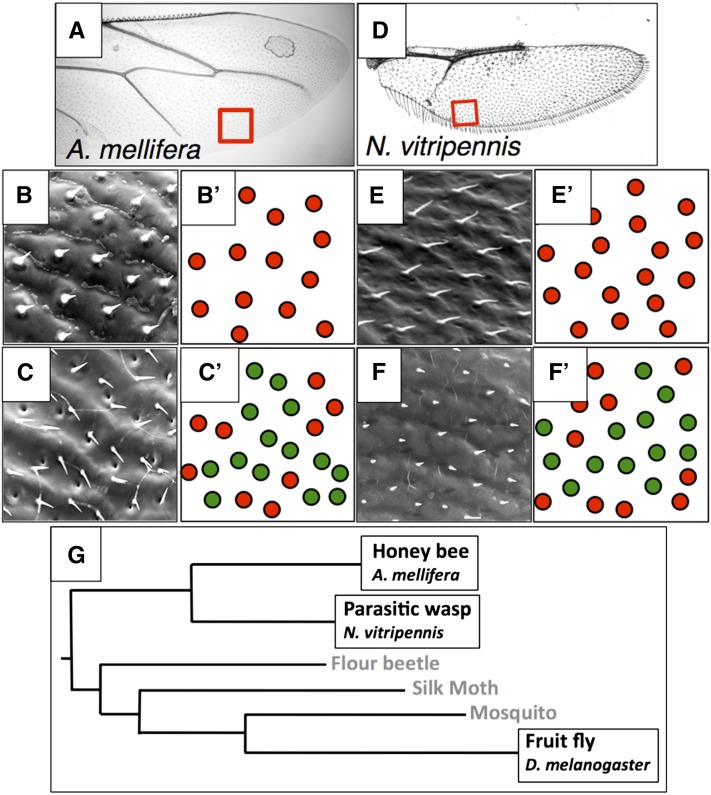
Hair position is coordinated with membrane topography on the dorsal but not ventral, surface of Hymenopteran wings. In all micrographs, anterior is uppermost and distal is to the right. For panels B′, C′, E′, and F′; red circles represent wing hairs that are positioned at, or near, the top of membrane ridges and green circles represent hairs that are positioned at, or near, the bottom of membrane troughs. (A) Light micrograph of honeybee hindwing. The unshaded red square indicates the region imaged in panels B and C. (B) SEM of dorsal surface of honeybee hindwing. (B′) Interpretation of panel B; dorsal hairs are positioned at the tops of wing membrane ridges. (C) SEM of ventral surface of honeybee hindwing. (C′) Interpretation of panel C; ventral hairs are located both on membrane ridges and within membrane troughs. (D) Light micrograph of Nasonia hindwing. The unshaded red square indicates the region imaged in panels F and G. (E) SEM of dorsal surface of Nasonia hindwing. (E′) Interpretation of panel E; dorsal hairs are positioned at the tops of wing membrane ridges. (F) SEM of ventral surface of Nasonia hindwing. (F′) Interpretation of panel F; ventral hairs are located both on membrane ridges and within membrane troughs. (G) Phylogenetic relationships between Apis and Nasonia (Hymenopterans), and Drosophila [adapted from (Savard *et al.* 2006)].

We have used SEM to image adult wings of two model Hymenopterans, the honeybee *A. mellifera* and the parasitic wasp *N. vitripennis*, to assess whether the coordination of hair position and membrane topography on the dorsal but not ventral wing is also a characteristic of the Hymenoptera. We have found that on a bee wing (Figure 3A), the stout hairs on the dorsal wing membrane are consistently located on the tops of membrane ridges (Figure 3, B and B′), whereas the locations of the fine hairs on the ventral wing are not coordinated with membrane topography (Figure 3, C and C′). Similarly, on a wasp wing (Figure 3D), dorsal hairs are at the tops of wing ridges (Figure 3, E and E′), but ventral hairs are not consistently positioned with respect to membrane topography (Figure 3, F and F′). Phylogenomic analyses have identified the Hymenopterans as basal within the Endopterygota family tree [Figure 3G (Savard *et al.* 2006)]. By this classification, the coordination of hair position and topography on the dorsal but not ventral wings of both model Hymenopterans suggests that the organization of wing membrane topography by the dorsal wing epithelium is a primitive characteristic of the Endopterygota.

**Figure 4:**
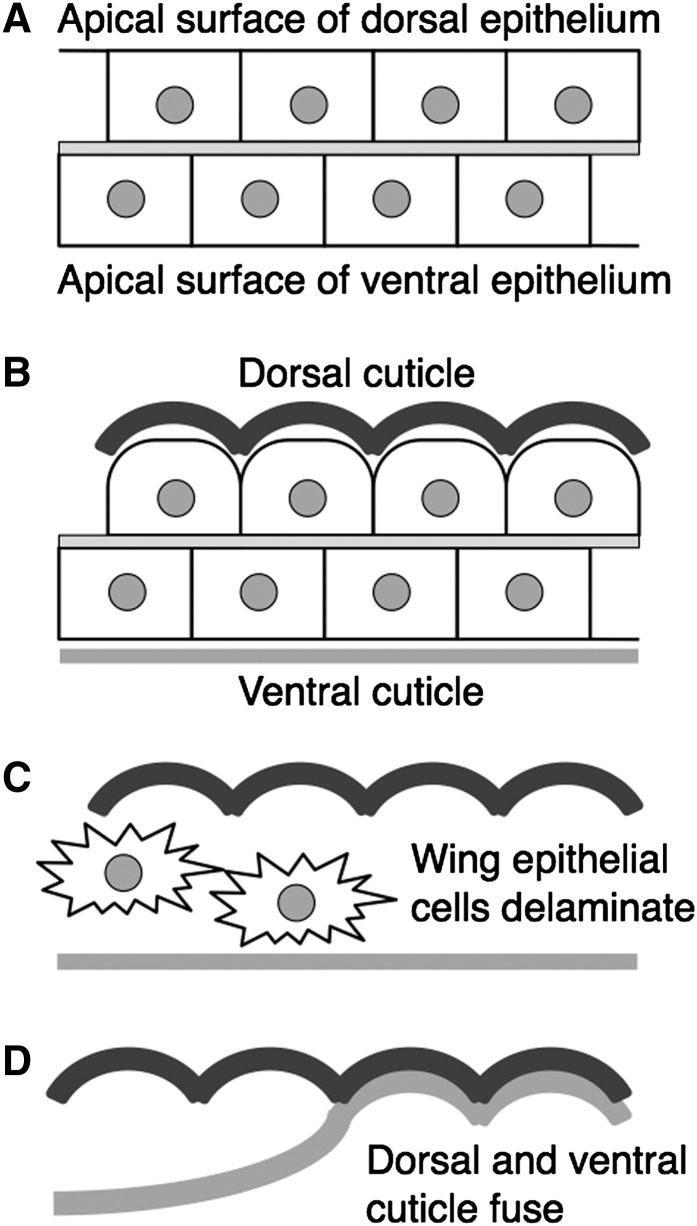
A Master-Slave model for insect membrane development adapted from Valentine and Collier (2011). (A) Dorsal and ventral pupal wing epithelia are attached at their basal surfaces. Note that the boundaries of dorsal and ventral cells are not aligned. (B) The apical surface of the dorsal epithelium acquires a ridged morphology prior to secreting rigid cuticle with a corresponding ridged topography. The ventral epithelium secretes more flexible cuticle. (C) Wing epithelial cells delaminate and leave the wing. (D) Dorsal and ventral cuticle fuse. The rigid dorsal cuticle acts as a mold to determine the shape of the flexible ventral cuticle.

Our observations in Dipterans and Hymenopterans have led us to propose a Master-Slave model for insect wing membrane development [Figure 4 and see (Valentine and Collier 2011)]. In the model, rigid cuticle, secreted by the dorsal wing epithelium, acts a mold to shape more flexible cuticle secreted by the ventral wing epithelium. A feature of the model is that it does not invoke cell signaling between dorsal and ventral wing cells, such as is known to modulate wing vein differentiation (Milan *et al.* 1997). Instead, the dorsal cuticle directly controls the morphology of the ventral cuticle after dorsal and ventral epithelial cells have delaminated and left the wing. The formation of a ridged dorsal cuticle is most likely due to a change in the apical morphology of dorsal wing cells prior to cuticle secretion. This seems to be the mechanism by which butterfly wing cells generate the elaborately shaped scales found on the adult wing membrane (Ghiradella 1994). In support of our Master-Slave model, a recent paper reports that the dorsal wing cuticle of the Hymenopteran Omphale is thicker than the ventral cuticle, and is the primary determinant of wing interference patterns that derive from wing membrane structure (Shevtsova *et al.* 2011).
